# Recent advancement of fisetin-based nanoformulations in the management of psoriasis

**DOI:** 10.1186/s11671-025-04298-4

**Published:** 2025-07-07

**Authors:** Umesh B. Telrandhe, Anjum N. Hasnain, Sachin N. Kothawade, Darshan R. Telange

**Affiliations:** 1 Datta Meghe College of Pharmacy, Datta Meghe Institute of Higher Education and Research (DMIHER) (DU), Sawangi Meghe, Wardha, Maharashtra 442001 India; 2 SCSSS’s Sitabai Thite College of Pharmacy, Near Pune-Nagar Bypass, Behind Hudco Colony, Shirur, Maharashtra 412210 India

**Keywords:** Psoriasis, Fisetin, Nanoformulations, Mechanism of action, Clinical and preclinical

## Abstract

Psoriasis is a chronic inflammatory autoimmune skin disease with enhanced skin cell turnover. Despite the therapies currently available, better and target-oriented therapies are needed. Fisetin is a flavonoid with antioxidant, anti-inflammatory, and immunomodulatory properties. It shows therapeutic potential, but its poor bioavailability and penetration into the skin cannot be used effectively to treat psoriasis. While fisetin–loaded nanoformulations in cancer and other diseases have been explored, their potential as a therapy for psoriasis is unexplored. Most reviews detail the biological activities of fisetin or nanoformulations for psoriasis therapy but not their combination. The review here compiles fisetin’s chemical and pharmacological properties along with the problems with conventional drug delivery and fisetin-loaded nanoformulations such as polymeric nanoparticles, liposomes, solid lipid nanoparticles, nanogels, and micelles. It also discusses their mechanisms, preclinical results, and potential for the clinic. Preclinical studies demonstrate fisetin nanoformulations to enhance penetration into the skin, reduce inflammation, promote skin regeneration in psoriasis models, and alleviate symptoms of redness and scaling. Clinical trials are lacking, and studies are needed to assess safety and efficacy. Fisetin nanoformulations are a potential target-oriented psoriasis therapy with better drug delivery and fewer side effects than conventional therapies. Despite formulation stability, scalability, and regulatory issues, the potential for fisetin-loaded nanoformulations is excellent and needs further exploration for their safety and efficacy in patients.

**Graphical abstract:**

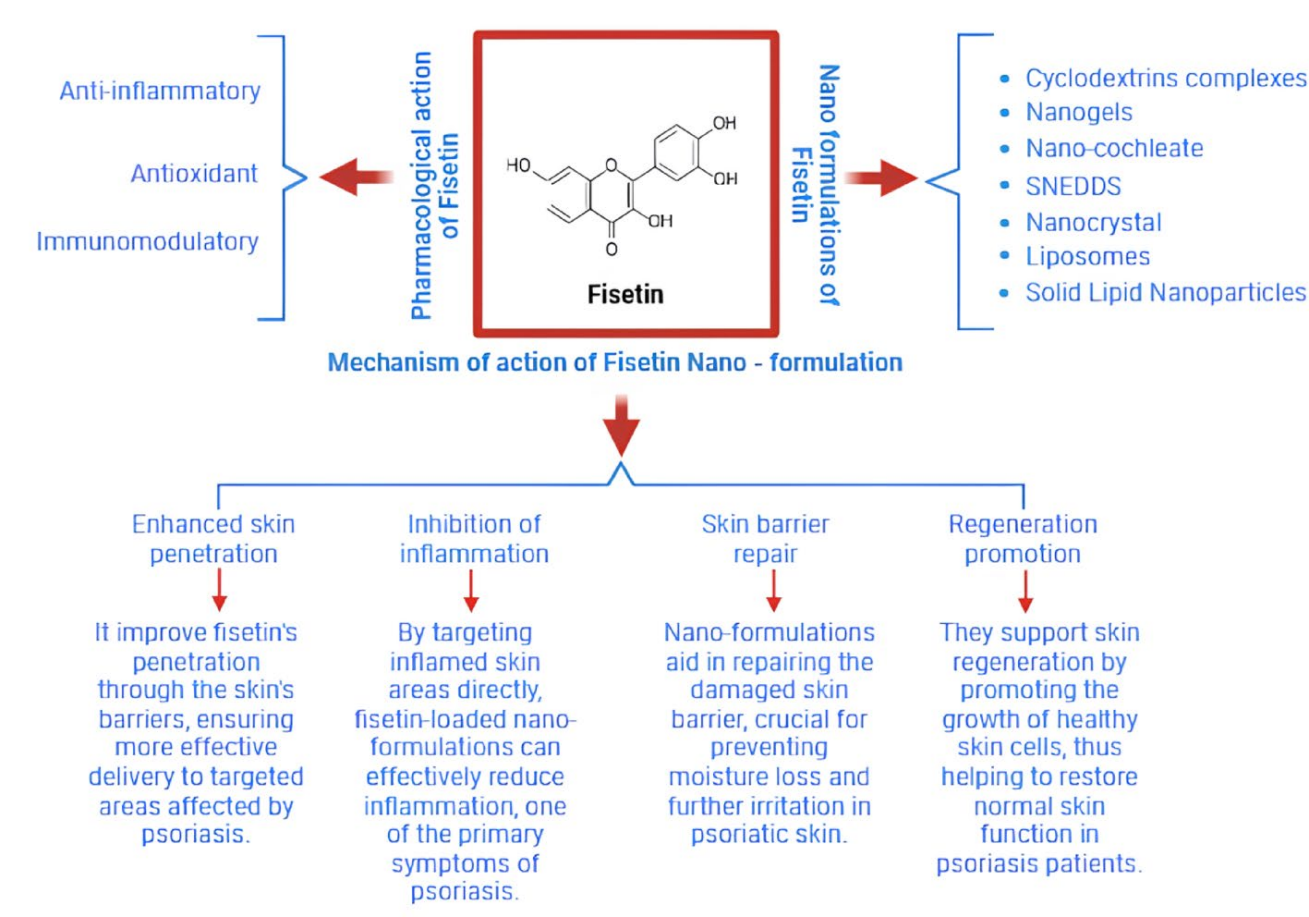

## Introduction

Psoriasis (PsO) is an autoimmune disorder that causes a good growth of the skin cells, forming red, scaly patches [1]. Patches typically appear on the elbows, the knees, the lower part of the back, and the scalp, but can appear on any part of the body. PsO has variable severity, ranging from small patches to large patches of red, inflamed skin [2]. The etiology of PsO has not yet been fully explained, but has been conceived as due to an overactivated immune system that causes an overgrowth of the skin cells [3]. Environmental factors, including infections, stress, or skin injury, play a crucial role in the triggering of eruptions of PsO [4]. There are different types of PsO, the most common being plaque psoriasis, also known as psoriasis vulgaris, characterized by raised, red patches covered with silvery-white scales accounting for 80–90% of cases [5]. Other types include guttate psoriasis (small, drop-shaped lesions), found more commonly in children and young adults and represents less than 2% of psoriasis cases [2], inverse psoriasis, also known as intertriginous or flexural psoriasis, affects 3–7% of psoriasis patients (smooth, red patches in skin folds) [3], and psoriatic arthritis (a form of arthritis affecting joints). PsO is often accompanied by itching, discomfort, and pain, which can significantly impact patient's quality of life.

Managing PsO is challenging because there is no known cure for the condition, and treatments aim primarily at controlling symptoms and reducing eruptions [4]. The main treatment approaches include topical therapies (like corticosteroids and vitamin D analogs), [5, 6] phototherapy (using ultraviolet light to slow skin cell growth), [6] and systemic treatments (oral or injectable medications that affect the immune system) [7]. These treatments, nevertheless, come with their own list of problems. Topical treatments may be acceptable for mild cases but not severe ones, and extended use of corticosteroids causes side effects, such as thinning of the skin [8]. Phototherapy, too, is a laborious process, with repeated sessions at the clinic, and systemics, though efficient, come with severe side effects, including suppression of the immune system, damage to the liver, or increased susceptibility to infections [6]. Therefore, new therapeutic agents that are as efficient but safer for long-term use are increasingly necessary. PsO is a T cell-mediated inflammatory disease that is driven by a cytokine imbalance that accelerates keratinization. While normal keratinization, the process by which basally located keratinocytes are converted to anucleate corneocytes, takes about 50 days, but in the case of psoriasis, it takes only 5 days, causing accelerated epidermal turnover and the formation of scaly plaques [9]. PsO pathophysiology includes a cascade of interrelated processes. Keratinocyte hyperproliferation leads to an excessive buildup of keratin, forming thick, scaly plaques in the stratum corneum. It is accompanied by the dilation and proliferation of dermal blood vessels, which enhances local inflammation.

**Fig. 1:**
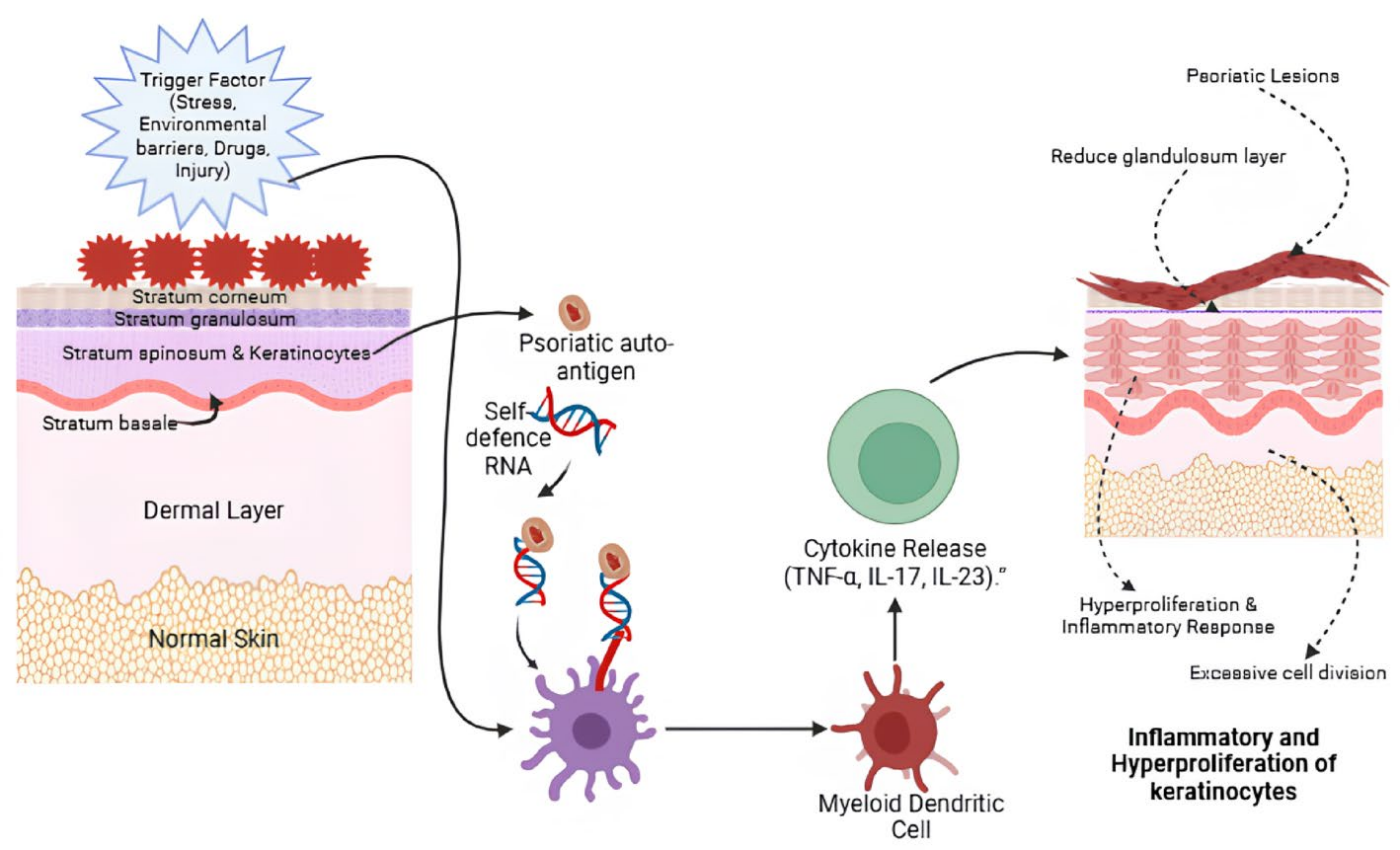
Pathophysiology of psoriasis disease

Additionally, there is a significant accumulation of inflammatory cells and neutrophils, along with a marked decrease or complete absence of granular tissue, all of which disrupt standard skin architecture and function [10]. Although the precise sequence of events leading to psoriasis remains unclear, current evidence suggests that various triggers and key abnormalities contribute to its development in genetically predisposed individuals. These factors disrupt normal skin homeostasis, setting off a cascade of inflammatory and cellular events that manifest as the clinical features of psoriasis [11]. The possible pathophysiological mechanisms underlying the disease are illustrated in Fig. 1.

The inception of psoriasis begins when provocative factors such as trauma, infection, drugs, stress, or physical injury trigger the skin, leading keratinocytes to release the psoriatic autoantigen LL37, an antimicrobial peptide. LL37 complexes with self-DNA activate plasmacytoid dendritic cells (pDCs) via TLR9, breaking self-tolerance and initiating a cascade of events. Activated pDCs secrete interferon (IFN), activating myeloid dendritic cells (mDCs). These mDCs produce cytokines such as IL-12, IL-23, and TNF, promoting the differentiation of naïve T-cells into TH1, TH17, and TH22 subsets. These T-cells migrate to the epidermis and release additional cytokines such as TNF, IFN, IL-17A, IL-17F, and IL-22, amplifying the inflammatory cascade and driving abnormal keratinocyte proliferation. Furthermore, overexpression of angiogenic mediators, including vascular endothelial growth factor (VEGF), TNF, IL-8, and IL-17, further accelerates the development of psoriatic plaques [12].

### Role of Fisetin as a therapeutic agent

Fisetin (FTN), a naturally occurring flavonoid [13], has also been found to be a strong antioxidant [14], anti-inflammatory agent [15], immunomodulator [16], and anticancer agent [17] that places it in a position to be a therapeutic agent in the treatment of many diseases, including PsO. One of the primary concerns in the treatment of PsO is the resultant inflammation on the skin. The anti-inflammatory property of FTN has the potential to suppress the inflammation by inhibiting the activity of some immune cells that make the skin be over-activated in response [18]. FTN has been proven to modulate the activity of the immune system, which shall be expected to suppress the hyperactivated immune system of PsO. Inhibiting the immune cells that cause the proliferation of the skin cells, FTN has the potential to inhibit the characteristic scales of the psoriasis due to enhanced skin turnover. Apart from the anti-inflammatory effects of FTN, it also has antioxidant activities that protect the skin against oxidative stress, which results in inflammation and skin aging. FTN protects skin cells against injury by eliminating poisonous free radicals and induces better-appearing skin. It would greatly benefit PsO patients, whose skin cells are constantly damaged due to the disease’s high turnover and inflammatory processe [19]. Moreover, FTN helps improve skin healing and prevent fibrosis, a common problem in chronic PsO [20]. Some studies suggest FTN prevents PsO from spreading to healthy skin parts, possibly better-managing eruptions [21]. FTN is a natural phytoconstituent; it can be a better option with fewer side effects than other complementary treatment alternatives because they are more aggressive [22]. Among the many natural flavonoids, FTN is noteworthy due to its multi-faceted pharmacological profile and higher efficacy in the treatment of psoriasis. In comparison to other flavonoids such as quercetin, kaempferol, or apigenin, FTN is more effective in the inhibition of cytokines like TNF-α, IL-6, IL-17, and IL-23, which are key to the pathogenesis of psoriasis. It targets both the inflammatory and proliferative processes such as NF-κB and STAT3 uniquely, in addition to antioxidant defense and normalization of keratinocytes. Moreover, FTN is more stable and compatible with the use of a nanocarrier, advancing its skin penetration and therapeutic effects in topicals. These properties make FTN a good natural flavonoid for the treatment of psoriasis in comparison with other flavonoids.

This review bridges the gap with the literature as the majority either reviews the biological effects of FTN alone or reviews nanoformulations for PsO without combining the two. The combination of the bioactivity of FTN with the latest advances in nanotechnology provides the review with insights into how nanoformulations can overcome the poor penetration and low bioavailability of FTN into the skin. Polymeric nanoparticles, liposomes, and solid lipid nanoparticles are the nanoformulation approaches employed for improving drug delivery and effectively targeting the layers of the skin with the advantages of sustained release, target action, and reduced side effects. The hydrophobicity of FTN, reflected by its relatively high log *P* value (~ 3.1), favors its retention in the skin layers when applied topically, making it suitable for localized psoriasis treatment. However, its poor aqueous solubility and low oral bioavailability pose challenges for systemic delivery routes such as oral or IV administration. Therefore, nanocarriers are designed not only to enhance skin penetration but also to improve pharmacokinetic parameters—like solubility, absorption, and sustained release—depending on the route of administration. While the preclinical findings are promising for nanoformulations with FTN, the clinical data are limited, which inhibits their actual usage and necessitates further studies. In summary, this review incorporates recent developments in the nanoformulations of FTN, focusing on their therapeutic potential against PsO and their need for clinical testing. The future focus should be optimizing formulation stability, scalability, and regulatory acceptability to translate the results into PsO therapies.

## Fisetin: a bioactive compound

**Fig. 2:**
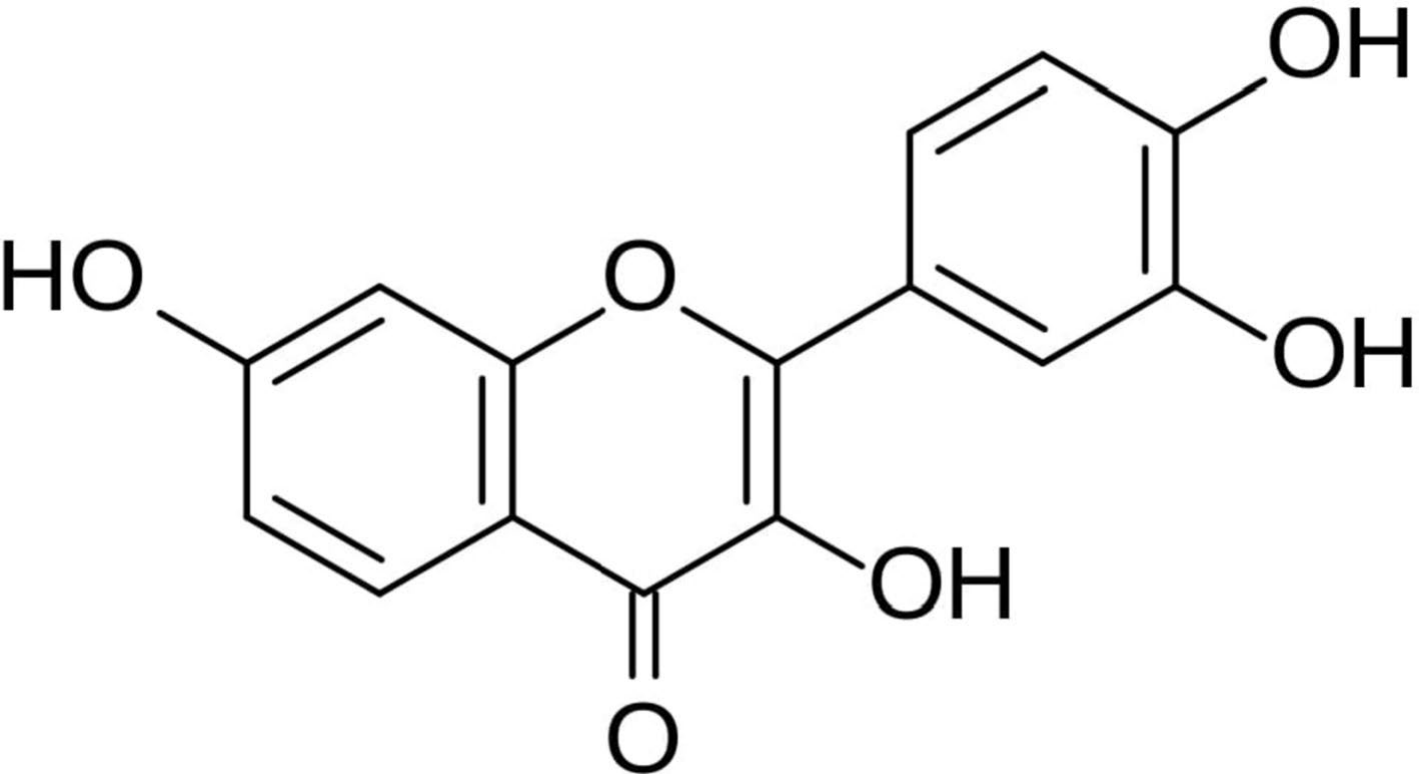
Molecular structure of FTN

**Fig. 3:**
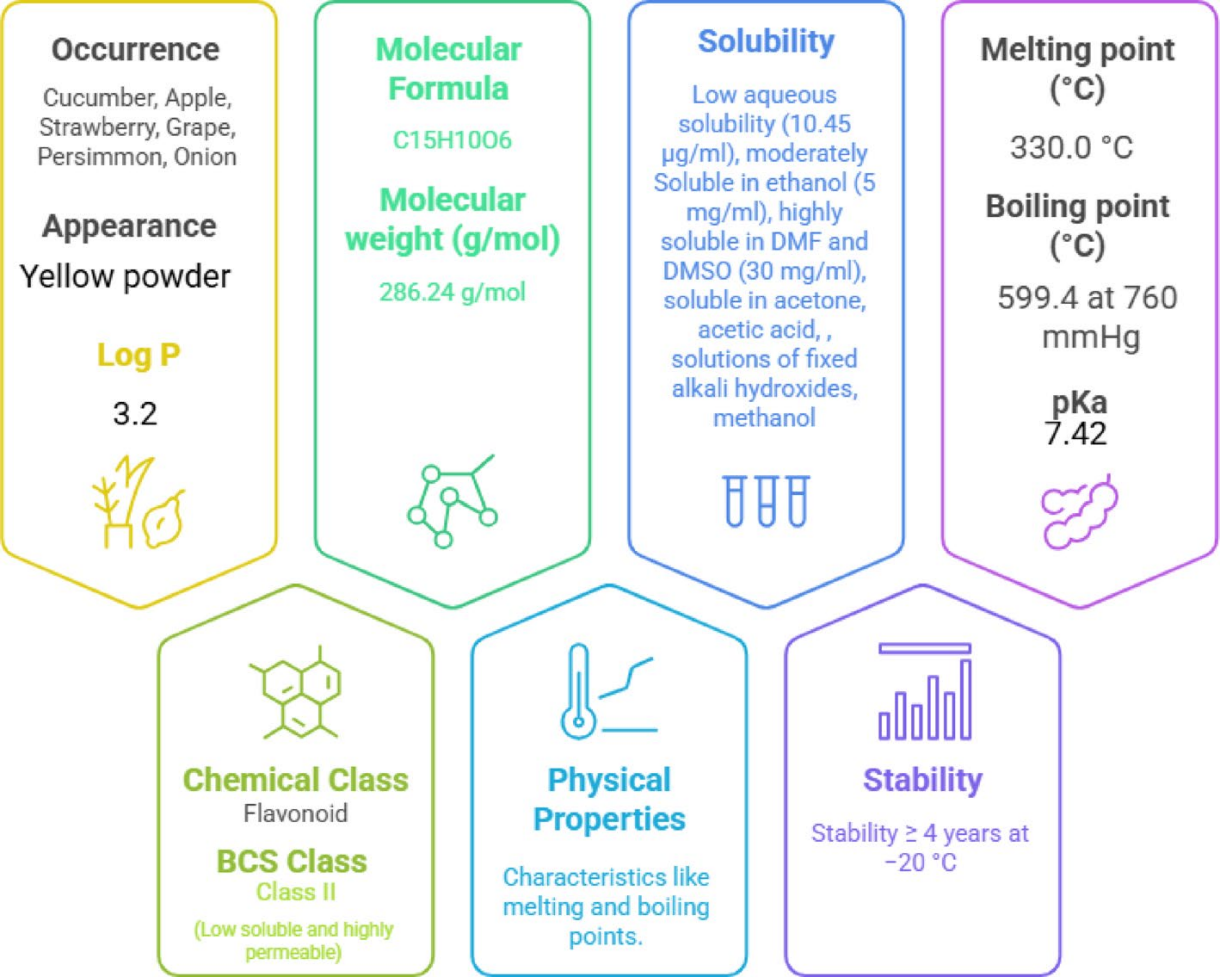
Physicochemical properties of FTN

FTN is a type of flavonoid, a class of natural polyphenolic compounds found in many fruits and vegetables, including strawberries, apples, persimmons, mangoes, grapes and kiwis, tomatoes, onions, cucumbers, etc. They act as a coloring agent [22]. The levels of FTN range from 2 to 160 μg/g in various fruits and vegetables, with the average daily intake estimated to be around 0.4 mg per individual. It has a simple chemical structure (Fig. 2) composed of a core of three benzene rings (phenyl groups) and hydroxyl (OH) groups attached to it [23]. This structure gives FTN its antioxidant and anti-inflammatory properties, as the hydroxyl groups allow it to interact with various molecules in the body, neutralizing harmful free radicals and modulating immune response [24]. FTN is water-soluble but has low bioavailability, meaning it doesn’t quickly enter the bloodstream or tissues in large amounts when taken orally [25]. Its physicochemical properties are given in Fig. 3.

This limitation can affect its effectiveness in treating conditions like psoriasis, where it needs to reach the skin or systemic circulation to have a therapeutic effect.

### Pharmacological activities relevant to PsO

FTN has attracted attention due to its interesting biological activity, including anti-inflammatory activity, antioxidant properties, antitumor activity, Alzheimer’s disease, and other pharmacological effects. FTN**,** has demonstrated various pharmacological activities that may be relevant to treating psoriasis (Table 1). FTN exhibits multiple mechanisms that make it effective for managing psoriasis. It reduces inflammation by inhibiting key molecules like cytokines and enzymes (e.g., NF-kB, COX-2), calming the overactive immune response [18, 26]. Its antioxidant properties help neutralize free radicals, reducing oxidative stress and skin damage [27]. It also supports skin protection and healing by promoting the regeneration of healthy skin cells and preventing scarring [28]. It modulates immune responses by regulating activated immune cells, restoring balance, and reducing skin lesions. Lastly, FTN has anti-proliferative effects, slowing down the rapid skin cell turnover in psoriasis [29], which helps prevent the formation of thick, scaly plaques.

**Table 1:** Mechanisms and action of FTN nanoformulations in PsO

Pharmacological activity	Mechanism of action	Effect on psoriasis	References
Anti-inflammatory	Inhibition of proinflammatory cytokines (TNF-α, IL-6, IL-1β), suppression of NF-kB and MAPK pathways	It helps to reduce inflammation and inhibits NF-κB signaling in PsO lesions	[30, 31]
Immunomodulatory	Modulation of T cell activity, suppression of Th17 cell responses, and reduction in IL-17 expression	FTN modulates immune responses by regulating T-cell activation and Th1/Th17 cytokine production which is a hallmark of psoriasis	[21, 32]
Antioxidant	Reduction of oxidative stress by scavenging free radicals and increasing antioxidant enzymes (SOD, CAT)	Fisetin scavenges reactive oxygen species (ROS) and reduces oxidative stress, which is elevated in psoriasis	[33, 34]
Anti-angiogenic	Inhibition of angiogenesis via suppression of VEGF and VEGFR signaling	FTN can influence blood vessel formation, reducing abnormal angiogenesis in PsO skin lesions Improves microcirculation in psoriatic skin and reduces redness	[21, 35]
Anti-proliferative	Inhibition of keratinocyte proliferation through cell cycle regulation and apoptosis induction	FTN can inhibit the hyperproliferation of keratinocytes, a key feature of PsO It helps reduce excessive skin cell turnover and scaling	[10, 20]
Antibacterial	Potential antibacterial activity, reducing the risk of infections associated with psoriasis lesions	Reduces secondary infections and inflammation in lesions	[36]
Wound healing	Enhanced collagen synthesis and promotion of wound healing, potentially aiding in psoriatic lesion repair	Reduces inflammation, promotes keratinocyte normalization, enhances tissue regeneration	[37]
Skin Barrier Restoration	FTN promotes the regeneration of the skin barrier by modulating epidermal differentiation and restoring barrier integrity	It improves skin hydration and reduces scaling and dryness in PsO	[20, 21]
Anti-apoptotic Activity	FTN prevents premature cell death (apoptosis) in skin cells, particularly keratinocytes, aiding tissue repair	Promotes healing and prevents further skin damage in PsO	[21, 31]

### Limitations of conventional Fisetin delivery

FTN showed immense potential in curing PsO [20, 21] but was hampered in its potential for treatment by various challenges in its delivery and efficacy. One of these challenges is poor bioavailability [30, 32] when taken orally, i.e., a small percentage of FTN is taken up in the bloodstream or psoriasis-inflicted body parts. It significantly reduced its potential to produce desired curative effects. Further, poor penetration of FTN in the skin restricts it from being given sufficiently to PsO lesions in local treatment, decreasing its efficacy when applied in creams, lotions, or patches [35, 37]. Further, FTN is unstable [17] and degrades when exposed to light, air, or heat, compromising the formulation and decreasing long-term efficacy [38].

A further problem is the low number of options for FTN formulations. Traditional methods, i.e., oral tablets and creams applied topically, failed to deliver appropriate concentrations to the desired location, particularly in more advanced cases of PsO [39]. While FTN was generally safe, its long-term effects in human subjects were not yet confirmed, and potential side effects, particularly at higher doses, were not yet definitively established [40]. Co-administration of FTN with other drugs was therefore in need of more studies.

## Nanoformulation strategies for Fisetin delivery

Nanotechnology plays a crucial role in enhancing the delivery of drugs [40], especially those like FTN, which has low bioavailability and limited skin penetration. By reducing the size of drug particles to the nanometer scale (1 to 100 nm), nanotechnology improves drug solubility, stability, and bioavailability [41, 42]. It allows for creating of advanced drug delivery systems that can overcome barriers like the skin’s tough outer layer or poor oral absorption. In the case of FTN, the major obstacle to successful therapy is the low oral bioavailability (44.1%) [43] due to its poor aqueous solubility (10.45 μg/ml) [27] and high lipophilicity (log P 3.2) [43]. Nanotechnology-based formulations can improve their ability to reach target tissues, such as the skin, in PsO treatments. They provide controlled, sustained drug release, ensuring more effective and consistent therapeutic effects.

### Types of Fisetin-based nanoformulations

**Fig. 4:**
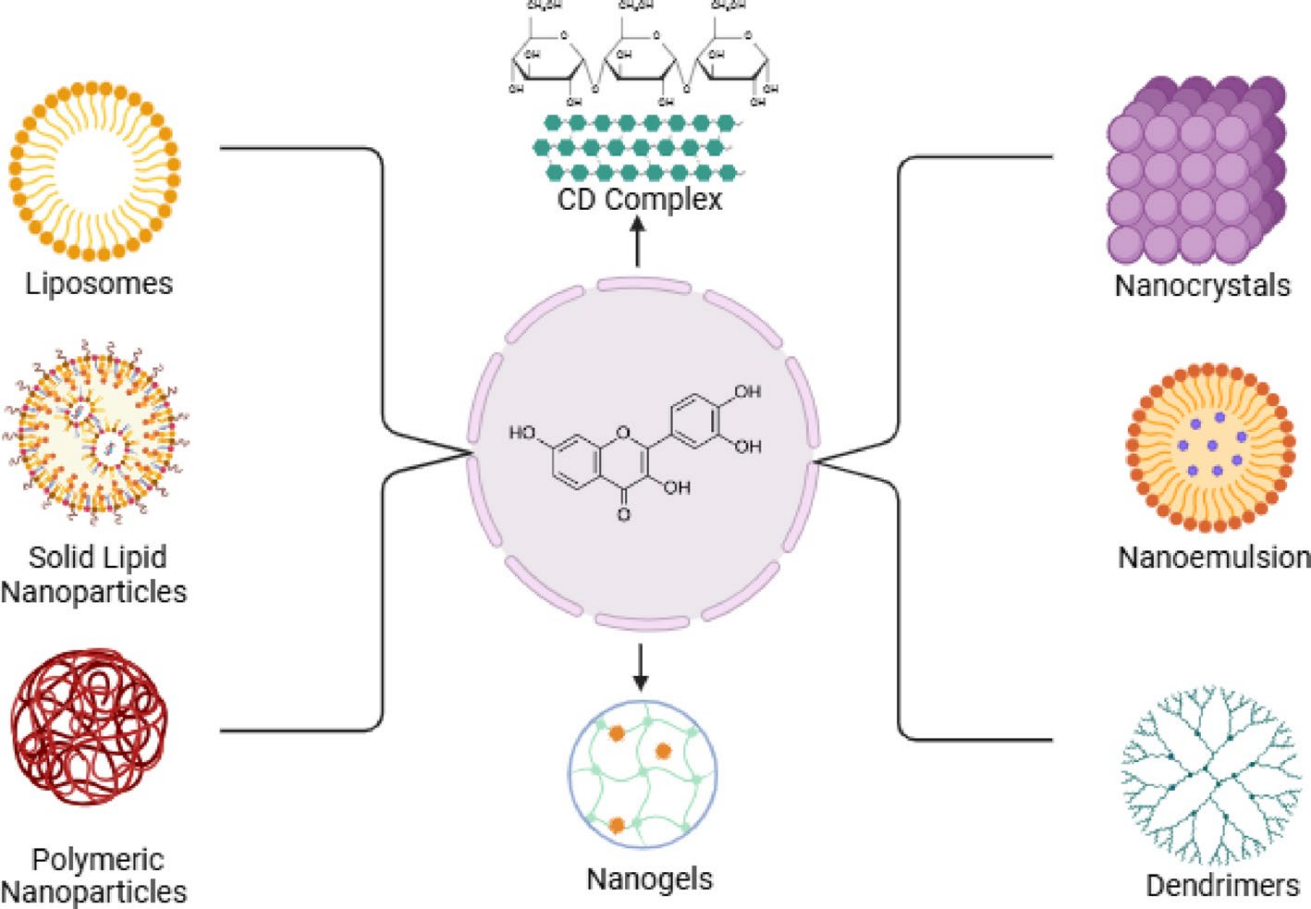
Types of FTN-Based Nanoformulations

Several nanoformulation strategies can be used to improve FTN delivery, including solid lipid nanoparticles, liposomes, nanocrystals, self-nano emulsifying drug delivery systems, nanocochleates, and polymeric nanoparticles, nanogel, and cyclodextrins complexes (Table 2 and Fig. 4). Each of these systems offers specific advantages for enhancing the absorption and effectiveness of FTN in treating conditions like psoriasis.

**Table 2:** Common nanoformulations strategies for FTN delivery

Nanoformulations strategy	Description	Advantages	Examples
Liposomes	Lipid-based vesicles that encapsulate FTN, enhancing its bioavailability	Biocompatible, improved solubility, controlled release, reduced toxicity	Liposomal Fisetin for oral or topical delivery
Solid lipid nanoparticles (SLNs)	Nanoparticles made of solid lipids for fisetin encapsulation	Stable, controlled release, good biocompatibility	SLN formulations for improved skin or oral delivery
Polymeric nanoparticles	Biodegradable polymers are used to encapsulate FTN, offering sustained release	Flexible formulation, improved stability, and bioavailability	PLGA (poly (lactic-co-glycolic acid)) nanoparticles
Nanocrystals	FTN in crystalline form with a reduced particle size to increase dissolution rate	Enhanced solubility, bioavailability, and rapid absorption	FTN-loaded nanocrystals for oral administration
Nanoemulsions	Oil-in-water or water-in-oil emulsions with FTN dissolved in the oil phase	Increased bioavailability, enhanced absorption, and stability	Nanoemulsion-based FTN for oral and topical applications
Dendrimers	Highly branched, nanoscale polymer structures that carry fisetin molecules	High loading capacity, precise control over release rates, and low toxicity	Dendrimers for targeted FTN delivery
Mesoporous silica nanoparticles	Silica-based nanoparticles with mesoporous structure for fisetin loading	High surface area, controlled drug release, and stability	Mesoporous silica nanoparticles for sustained FTN release
Magnetic nanoparticles	Iron oxide or similar magnetic materials to load FTN, enabling targeted delivery	Targeted delivery using magnetic field improved pharmacokinetics	Magnetic nanoparticles for targeted FTN therapy

#### Solid lipid nanoparticles

SLNs are colloidal carriers made by high-pressure homogenization of lipids, emulsifiers, and water, with particle sizes between < 100 and 1000 nm [44]. Their solid lipophilic matrix ensures controlled release and protects active molecules. Made of physiological lipids and devoid of organic solvents, SLNs have advantages like increased bioavailability for lipophilic drugs [44]. Researchers selected SLNs with a view to enhance the biological effect of FTN.

Kulbacka et al. (2016) designed a multifunctional delivery system with solid lipid nanoparticles (SLNs) co-loaded with FTN and the photosensitizer IR-780 in the proportion 5:1. The hydrodynamic diameter of the formulated SLNs was found to be 133.8 nm, and the efficiency of FTN encapsulation was found to be 41.19%. Phospholipid 90 G, cetyl palmitate, and Tween 80 were used to prepare the SLNs. In vitro experiments on hamster ovarian fibroblasts (CHO-K1) and human colon adenocarcinoma (LoVo) cells treated with electroporation prior to the experiment showed significant cell viability decrease (ca. 50%) as a result of photodynamic therapy [45]. The formulation also resulted in cytoskeleton rearrangement and up-regulation of p53 and manganese superoxide dismutase expression. The results set the ground for the promise of the multifunctional nanocarrier in cancer detection and therapy. Das et al. (2015) studied the photophysical properties of FTN in bulk aqueous medium and solid lipid nanoparticles (SLNs) in the pH range 5–9. Anionic species dominated in low pH, whereas the proton-transferred species dominated in the case of SLNs. In both media, the increased pH enhanced the emission of the anionic species. The results show that the efficiency of FTN as a drug could be a function of the neutral, anionic, and proton-transferred species in different pH [46]. These particles could be used to encase FTN and prevent degradation. SLNs also possess a plus point in the controlled release of drugs, which can boost the therapeutic efficacy of FTN in PsO. SLNs also allow for the penetration of FTN in the skin, making it penetrate psoriasis-inflicted layers of the skin. SLNs also stabilize FTN during storage, keeping it from degrading and offering a sustained therapeutic action. Since PsO is associated with oxidative stress and inflammation**,** understanding how FTN behaves in various pH conditions may help design targeted therapies for PsO skin, which has an altered pH balance.

#### Liposomes

Liposomes are a great delivery system for fat-soluble and water-soluble molecules since liposomes can hold them in different areas of the phospholipid bilayer [47]. Liposome bioactive molecules are protected from environmental harm and can be released in a desired place [48]. The surface functionalization of liposomes has allowed better skin penetration and transcutaneous permeation of anti-psoriatic drugs. Also, incorporating natural permeation enhancers in liposomes that manage the hydration status of the drug and/or skin has allowed better delivery of the drug to psoriatic skin [49]. The incorporation of natural enhancers in liposomes also allows better penetration of the drug into the skin. Liposomes also release the drug over time, eliminating multiple applications [50]. Mignet et al. (2012) developed a liposomal formulation of FTN to overcome its poor water solubility and enhance in vivo administration. Various preparation methods and lipid compositions were evaluated, leading to an optimized formulation using P90G and DODA-GLY-PEG2000. The resulting liposomes had a nanometer-scale diameter (175 nm), high homogeneity (PDI 0.12), and an FTN encapsulation efficiency of 73%. The formulation remained stable for 59 days and retained 80% of its FTN content by day 32. Additionally, liposomal FTN preserved the cytotoxic and morphological effects of free FTN in tumor and endothelial cell lines, making it a promising candidate for in vivo applications [51]. PsO treatments often require systemic or localized drug delivery**,** and liposomes can enhance penetration, prolong retention, and improve the bioavailability of FTN, making it more effective in reducing inflammation and keratinocyte hyperproliferation in PsO lesions.

#### Transethosome

Moolakkadath et al. optimized the topical delivery formulation of FTN using the Box-Behnken design for the transethosome formulation. The optimized formulation with a size of the vesicle as 74.21 ± 2.65 nm, the value of the zeta potential as − 11.0 mV, and the value of the entrapment efficiency as 68.31 ± 1.48% showed enhanced penetration through the skin. Confocal microscopy and dermatokinetic analysis provided deeper penetration when compared with hydroalcoholic solution. TEM analysis showed spherical, closed-upon-vesicles, while thermoanalytic techniques ensured fluidization of the membrane for better penetration. These findings suggest that the developed transethosomes formulation is a promising carrier for FTN in dermal drug delivery [52]. Since transethosomes enhance skin penetration**,** this formulation could be particularly beneficial for topical psoriasis treatment**,** ensuring more deep penetration into PsO plaques and improving anti-inflammatory and antioxidant effects**.**

#### Nanocrystal

Nanocrystal is aggregates of active drug molecules surrounded by a surfactant, typically smaller than 1 μm [53, 54]. Nanocrystals are not carrier material and become nano suspensions when dispersed in aqueous or non-aqueous media. They require stabilization using stabilizers or surfactants [55]. Methods employed to manufacture nanocrystals are milling, precipitation, homogenization, and spray-drying [56]. Nanotechnology, already affecting various fields like medicine and pharmacy, is revolutionizing drug formulation by increasing the surface area and dissolution rate of poorly soluble compounds, improving absorption from the gastrointestinal tract.

Dzakwan et al. developed an FTN nanosuspension using the nanoprecipitation technique to enhance its poor solubility and bioavailability. Various stabilizers were tested, with polysorbate 80 proving the most effective, resulting in nanosized particles (225.7 nm ± 1.31), a polydispersity index of 0.272 ± 0.02, and a zeta potential of -39.3 ± 0.26. TEM analysis confirmed the spherical morphology of the nanosuspension [57]. The improved solubility and stability of FTN nanosuspension make it a promising therapeutic candidate for PsO treatment. Given FTN’s potent anti-inflammatory and antioxidant properties, its enhanced bioavailability could help reduce oxidative stress, inflammation, and keratinocyte hyperproliferation associated with PsO.

Additionally, nanosuspension facilitates better skin penetration and drug retention, potentially improving the effectiveness of both topical and systemic PsO therapies. Ma et al. designed and characterized FTN nanocrystals stabilized with poloxamer P407, with nanoscale diameter (148.6 ± 1.1 nm), high homogeneity (PDI 0.17 ± 0.01), and production yield of 97.0%. The nanocrystals had good colloidal stability in various aqueous mediums and remained stable for at least 120 days when freeze-dried. Drug release experiments showed a cumulative release of 98.7% in three days, whereas in vitro experiments showed higher cytotoxicity and anti-angiogenic activity when compared with free FTN [58]. The higher bioavailability, stability, and solubility of FTN nanocrystals render it a promising candidate for PsO treatment. Their higher anti-angiogenic and anti-inflammatory action might suppress the abnormal growth of new vessels and the immunity-related responses involved in the formation of the psoriatic lesion. Additionally, the sustained drug release and higher cellular uptake might lead to more efficient and longer-acting therapeutic action, rendering nanocrystal-based FTN a promising therapeutic agent for topical and systemic PsO therapy.

#### Self-Nanoemulsifying drug delivery system (SNEDDS)

SNEDDS is a blend of oil, cosurfactant, and a surfactant with HLB > 12, which is anhydrous and isotropic in character. The process provided better control, repeatability, solubility, and stability, as well as better bioavailability of the active ingredient. The process also increased patient compliance and reduced the production costs [59]. Solid form of liquid SNEDDS provided better stability and patient compliance [60].

Kumar et al. designed a self-nano-emulsifying drug delivery system (SNEDDS) for FTN to enhance its low water solubility and low bioavailability. Pharmacokinetic evaluation showed that SNEDDS enhanced Cmax, area under the curve, and the mean residence time of FTN in the plasma significantly. In rotenone-induced model of Parkinson’s disease (PD) in rats, FTN-SNEDDS showed enhanced neuroprotective activity, as evidenced by enhanced locomotor activity, muscle coordination, and biochemical markers such as oxidative stress factors (TBARS, nitrite, GSH, SOD, CAT) and inflammatory cytokines (TNF-α, IL-6). These findings suggest that SNEDDS formulation enhances the therapeutic value of FTN significantly [61]. The increased bioavailability, water solubility, and anti-inflammatory activity of FTN-SNEDDS may be useful in PsO therapy. Since oxidative stress and chronic inflammation are critical factors in PsO pathogenesis, the increased absorption and sustained delivery of FTN-SNEDDS may lead to increased immune modulation, reduced inflammation, and increased therapeutic outcomes. FTN-SNEDDS could be an effective means for topical and systemic PsO therapy. Kumar et al. designed a self-nano emulsifying drug delivery system (SNEDDS) formulation for FTN which significantly enhanced rotenone-induced neurological impairments in rats, with better efficiency than naïve FTN in correcting body weight loss, grip strength, beam walk, and the level of dopamine. Histopathological analysis confirmed its neuroprotective activity, suggesting its potential for increased therapeutic benefits [62]. The increased bioavailability, water solubility, and therapeutic activity of SNEDDS-loaded FTN may be useful for PsO therapy as well. Since PsO is associated with chronic inflammation, oxidative stress, and dysregulated immunity, the augmented absorption and systemic delivery of FTN using SNEDDS may increase its anti-inflammatory and antioxidant activity.

This improved formulation could lead to more effective management of PsO symptoms and better drug penetration for topical and systemic applications.

SNEDDS can be used in transdermal patches to enhance the solubility and bioavailability of poorly water-soluble drugs, improving skin permeability and enabling controlled release for better absorption [63]. Although FTN shows potential benefits for keratinocyte damage, limited research exists on its topical application, with most studies focusing on intravenous or intraperitoneal doses. However, existing results suggest further exploration of its topical use.

#### Nanocochleates

Nanocochleates are cigar-shaped structures formed by lipid bilayers created by interacting negatively charged liposomes and cationic salts, typically calcium ions. Phospholipids are essential in their formation, serving as the core material [64]. Since nanocochleates are made from natural lipids, they offer enhanced stability under harsh environmental conditions, making them more advantageous than other nanoparticulate systems. This stability is crucial for the effectiveness of various drug delivery applications. More and more people are interested in Nanocochleates because they have benefits like better stability, protection, drug release over time, and better bioavailability of encapsulated pharmaceuticals [65]***.***

Bothiraja et al. (2014) developed FTN-loaded nanocochleates to enhance their therapeutic efficacy by converting FTN-loaded liposomal vesicles into nanocochleates using Ca^2^⁺ ions. The resulting formulation exhibited a particle size of 275 ± 4 nm, encapsulation efficiency of 84.31 ± 2.52%, and demonstrated stability, sustained drug release, and improved safety. In vitro studies showed a 1.3-fold increase in anticancer activity against MCF-7 cells, while pharmacokinetic studies in mice revealed a remarkable 141-fold improvement in relative bioavailability with low tissue distribution [66]. The increased bioavailability, stability, and controlled delivery of FTN as nanocochleates would be extremely valuable for PsO therapy. Since PsO is triggered by chronic inflammation, oxidative stress, and dysregulated immunity, the anti-inflammatory and antioxidant action of FTN could be better exploited through this delivery system. The ability of nanocochleates to increase the absorption of drugs and extend the retention of drugs could lead to sustained therapeutic action, fewer system-side effects, and enhanced penetration through the skin, making FTN-loaded nanocochleates a promising system for topical and systemic PsO therapy. Since nanocochleates are lipidic in nature and are capable of delivering hydrophobic as well as hydrophilic drugs, nanocochleates could be useful in the delivery of active compounds through the skin [63]. Their controlled drug delivery, drug protection in their inner compartment, and stability make nanocochleates suitable to be employed in transdermal applications. Nanocochleates, by increasing the permeability of drugs through the skin and their solubility, could make active ingredient uptake more efficient, providing a promising solution to transdermal drug delivery systems [64].

#### Nanogel

Nanogel comprises cross-linked hydrogels that are biocompatible and biodegradable, which are capable of holding water or drugs in a three-dimensional system [67]. The systems are highly biocompatible and are capable of delivering phytoconstituents in a way that facilitates better penetration through the skin as well as hydration. Nanogels can provide a controlled release of FTN, which helps manage chronic diseases such as psoriasis and is desired to generate effects that endure over time [67]. The gel-like consistency also allows for easy application to the skin, improving patient adherence to treatment.

Singh et al. (2024) investigated the efficacy of nano gel from the extract of *Heydotis corymbosa* for enhanced permeation and prolonged deposition in psoriasis-like dermatitis. Optimized nanophytosomes were loaded in pluronic gel, with a spherical shape having 73.05% entrapment efficacy, size 86.11 nm, and − 10.40 mV zeta potential. In ex vivo and in vivo experiments, anti-psoriatic activity of the nanogel was established with enhanced skin integrity and reduced inflammatory cytokines in a rat model. These results prove that the phytoconstituents -based nanogel effectively alleviates PsO with enhanced skin retention [68]. Ahmad A. et al. suggested that curcumin nanogel preparations can enhance bioavailability,stability and reduce the required dosage of curcumin for PsO treatment. It highlights curcumin nanogels as a promising, more effective, and safer alternative to traditional PsO treatments [69]. Deghiedy et al. (2024) investigated the potential of FTN-loaded Pluronic-PAMPS (PLUR-PAMPS) nanogels in managing Alzheimer’s disease (AD). AD was induced in rats using aluminum chloride (AlCl3) and D-galactose (D-gal) [70]. As FTN has low bioavailability, PLUR-PAMPS nanogels were fabricated using gamma radiation to enhance their stability and solubility. Male Wistar rats were administered FTN or FTN-PLUR-PAMPS before the administration of AlCl3/D-gal. Behavioral, histopathological, and biochemistry tests showed that FTN-PLUR-PAMPS successfully relieved cognitive impairment and neurodegeneration. The findings suggested that nanogel-loaded FTN increased bioavailability and therapeutic efficacy, which has potential as a new AD therapeutic method [70].

#### Cyclodextrins complexes

Cyclodextrins (CD) are stable enzymatic degradation products that possess a hydrophilic outer face and a lipophilic inner core. They are effective molecular chelating agents with a lipophilic inner core and hydrophilic outer face. CDs, due to their unique structure, are capable of encaging hydrophobic molecules, increasing their aqueous solution stability and water solubility [71]. Naturally occurring CDs most commonly utilized are α-CD, β-CD, and γ-CD [72]. CD complexes form a good base to develop FTN new formulations with preferred properties.

Pais et al. obtained an FTN/γ-CD (1:1) inclusion complex using co-dissolution and subsequent co-lyophilization. γ-CD incorporated the FTN molecule more efficiently with less rearrangement, which allowed for better packing in the channels. The obtained complex also demonstrated satisfactory antioxidant activity in the DPPH assay [73].

Kadari et al. prepared an FTN-hydroxypropyl-β-cyclodextrin (HP-β-CD) complex using coacervation and then encapsulated it into PLGA nanoparticles (NPs) via the multiple emulsion method. The NPs had a size of 87.27 ± 0.10 nm, negative zeta potential (− 8.71 ± 0.03 mV), and 96.32% yield. They displayed a biphasic release, with 43% of the drug released in 8 h and sustained release for 72 h. FTN-NPs showed 3.9 times higher cytotoxicity against MCF-7 cells and enhanced intracellular delivery. In vivo studies revealed improved bioavailability and mean residence time for oral FTN-NPs compared to FTN suspension [74].

## Mechanisms of action of FTN nanoformulations IN PsO

FTN nanoformulations offer significant potential for treating PsO through various mechanisms. The key mechanisms through which FTN works include its anti-inflammatory effects, immunomodulatory properties, antioxidant activity, and ability to restore the skin barrier (Table 1). These properties are enhanced when FTN is delivered in nanoformulations, as they improve its water solubility, absorption, stability, targeted action, and bioavailability.

### Anti-inflammatory effects

PsO is caused predominantly by excessive inflammation in the skin, triggered by overactive immunity [75]. FTN, with potent anti-inflammatory, antioxidant, and immunomodulatory properties, shows promise for PsO treatment, though its low bioavailability limits clinical use. Nanoformulations such as nanogels, nanoparticles, liposomes, and micelles enhance its stability, solubility, and targeted skin delivery. Immune cells like T-cells produce proinflammatory molecules that lead to excessive turnover of skin cells and scaly lesions in this condition [76]. FTN nanoformulations mitigate PsO by suppressing the NF-κB pathway, reducing proinflammatory cytokines (TNF-α, IL-6, IL-1β), and downregulating the IL-23/Th17 axis, thereby controlling keratinocyte hyperproliferation [20]. They also activate Nrf2 to combat oxidative stress, inhibit the mTOR/AKT pathway to reduce epidermal hyperplasia, restore skin barrier function by improving ceramide synthesis, and lower COX-2 and PGE2 expression, alleviating inflammation [77]. These nanoformulations provide sustained therapeutic effects, making them a promising strategy for PsO management.

### Immunomodulatory properties

In PsO, there is excessive activation of the immune system, and T-cells and dendritic cells get inappropriately activated, resulting in excessive cell replication in the skin [78, 79]. The immunomodulatory activity of FTN inhibits excessive activation of these immune cells. When flavonoid is given in nanoformulations, it is better able to penetrate the skin and migrate to immune cells in diseased tissues [80]. FTN controls signals that activate immune function, restoring a balance to the immune system [81]. In this manner, FTN inhibits autoimmunologic attacks on the skin, slowing the excessive turnover of skin cells that lead to scaly psoriasis plaques. In addition to acting on immune cells, FTN also regulates other molecules involved in immunity, such as NF-kB (protein complex that controls many of the genes that produce proinflammatory effects) and TNF-α (cytokine that stimulates inflammation). FTN reduces the immune system’s function and PsO symptoms by inhibiting these mechanisms.

### Antioxidant activity

Oxidative stress, caused by a disequilibrium between antioxidants and free radicals, is a primary reason for PsO progression and worsening [82]. Free radicals can be harmful to skin cells, irritating the skin, causing inflammation, etc. FTN is a potent antioxidant that inactivates such free radicals [14]. When FTN is presented in nanoformulations, its antioxidant activity is heightened because it is protected from degradation and can more easily penetrate the skin. By neutralizing free radicals in the skin, FTN minimizes oxidative damage and stimulates healthier skin cells. It is of special interest in PsO, in which oxidative stress induces inflammation and excessive skin cell turnover [83]. Furthermore, FTN’s antioxidant effects also reduce skin irritation, discomfort, and itching, all common psoriasis symptoms [84]. By inhibiting oxidative damage, FTN increases the skin’s health in general and prevents such damage from recurring, allowing the skin to heal more effectively [43].

### Skin barrier restoration

One of the significant challenges of treating PsO involves restoring the skin’s natural barrier. The skin’s natural barrier fails in psoriasis and leads to the drying and susceptibility of the skin to irritation and infection [85]. The nanoforms of FTN can be utilized to restore the skin’s natural barrier by triggering the repair of the skin cells and moistening them. It facilitates the generation of new skin cells while maintaining the topmost layer of the skin and keeping it in a state of wellness [86]. It is done by regulating the development and maturing of keratinocytes (cells of the topmost layer of the skin), restoring a functional and healthy natural barrier of the skin [87]. Applying FTN topically on the skin in nanoforms makes it better able to induce the repair of the skin, speeding the recovery and reducing the intensity of the drying and flakiness of the skin. FTN also increases the natural moistening of the skin, a natural process that generally becomes lacking in PsO [86]. This moistening effect not only calms the skin but also prevents skin irritation from drying and cracking. The nanoforms enable FTN to get the required skin depths, restore natural barrier function, and boost the skin’s long-term wellness.

Table 1 captures how fisetin nanoformulations may work at multiple levels to treat and manage PsO, from reducing inflammation to restoring the skin barrier.

## Preclinical studies on FTN-based nanoformulations

FTN, an anti-inflammatory flavonoid with antioxidant and anticancer activities, has been a subject of great interest in preclinical evaluations of the therapeutic application of nanoformulations. Its limited solubility in water and speedy metabolism restricted its effectiveness and prompted the need for nanotechnology-assisted drug-delivery systems with higher bioavailability stability and site-targeting therapeutic activities.

### In vitro studies on FTN nanoformulations

FTN nanoparticles have been widely studied in vitro in treating cancer, neuroprotection, and inflammation. Sundarraj et al. demonstrated that FTN encapsulated within the nanoparticles of poly(lactic-co-glycolic acid) effectively suppressed the oncogenic factor of critical heat shock factor 1 and released ROS that induced apoptosis of the cancer cells [88]. Similarly, Sharma et al. reviewed the flavonoid nanoparticle development. They demonstrated that FTN nanoparticles had higher cellular targeting and anticancer activities than free FTN against a panel of cancer cells [89]. Mehta et al. demonstrated in models of neurodegenerative disease that FTN lipid vesicles enhanced the viability of the neuron and relieved oxidative stress in models of Alzheimer’s disease [41]. In PsO, Chamcheu et al. demonstrated that FTN solid lipid nanoparticles and transferases had enhanced anti-inflammatory activity and suppressed keratinocyte growth in in vitro models of PoS [20].

### In vivo studies on FTN nanoformulations

Several in vivo models using animals have validated the efficacy of FTN nanoformulation in the therapy of cancer, neurodegenerative diseases, psoriasis, and metabolic disorders. Talaat et al. investigated PEGylated cholephytosomes for FTN delivery in breast cancer and found that the formulation significantly reduced tumor size and enhanced apoptotic markers in mice [90]. Likewise, Talaat et al. demonstrated that bio-inspired lipid nanoparticles improved the anti-tumoral efficacy of FTN in breast cancer xenografts [31]. In neurological disorders**,** Rakshit et al. examined nanoformulated FTN for Alzheimer’s disease treatment**,** reporting reduced proinflammatory cytokines and activation of the NRF2/HO-1 pathway, leading to neuroprotection in mice [34]**.** Mahawar et al. showed that FTN-loaded chitosan nanoparticles alleviated neurobehavioral alterations in a pilocarpine-induced epilepsy model**,** indicating its potential as an anticonvulsant therapy [91].

For PsO treatment**,** Awadeen et al. developed lipid-polymer hybrid nanoparticles (LPHNPs) for FTN and demonstrated their efficacy against severe acute pancreatitis and psoriasis-like skin inflammation in animal models [92]. In anti-aging studies**,** Krishnakumar et al. (2022) investigated a hybrid-hydrogel formulation of FTN**,** showing enhanced bioavailability and improved pharmacokinetics in aged mice models [25]. Nanoencapsulation improves drug solubility, stability, and bioavailability**,** allowing for targeted and sustained release**.** Comparison of in vitro vs. in vivo findings on FTN nanoformulations where shown in Table 3. Future clinical trials are essential to confirm these promising in vitro and in vivo results and to develop safe and effective FTN-based nanoformulations for human use**.**

**Table 3:** Comparison of *n itro* s. *n ivo* findings on FTN nanoformulations

Criteria	in vitro studies	in vivo studies
Study models	Cancer cell lines, neuronal cells, keratinocytes, fibroblasts	Animal models (mice, rats, xenografts)
Therapeutic targets	Cancer, neurodegeneration, psoriasis, inflammation	Tumor regression, neuroprotection, wound healing, metabolic disorders
Findings in cancer	Induces apoptosis via ROS generation and HSF1 inhibition [88] Enhanced cytotoxicity against breast and oral cancer cells [61]	Significant tumor reduction in breast cancer models using PEGylated nanoparticles [90] Improved bioavailability and antitumor effects in xenograft models [31]
Findings in Neurological Disorders	It enhances neuronal survival and reduces oxidative stress [41] Modulates PI3K/Akt/mTOR pathways for neuroprotection	Neuroprotection and cognitive improvement in Alzheimer’s models via NRF2/HO-1 activation [34] Reduces seizures in epilepsy models [91]
Findings in inflammatory skin disorders (Psoriasis)	Fisetin-loaded transfersomes and SLNs suppress keratinocyte proliferation [20]	Reduced inflammation and enhanced skin penetration in psoriasis models [92]
Bioavailability & Drug stability	Improved solubility, uptake, and stability in nanocarrier-based delivery	Enhanced systemic absorption and controlled release, reducing dosing frequency
Challenges & limitations	Limited predictive value due to lack of physiological complexity	Requires dose optimization and long-term safety validation

## Clinical potential of FTN nanoformulations

**Fig. 5:**
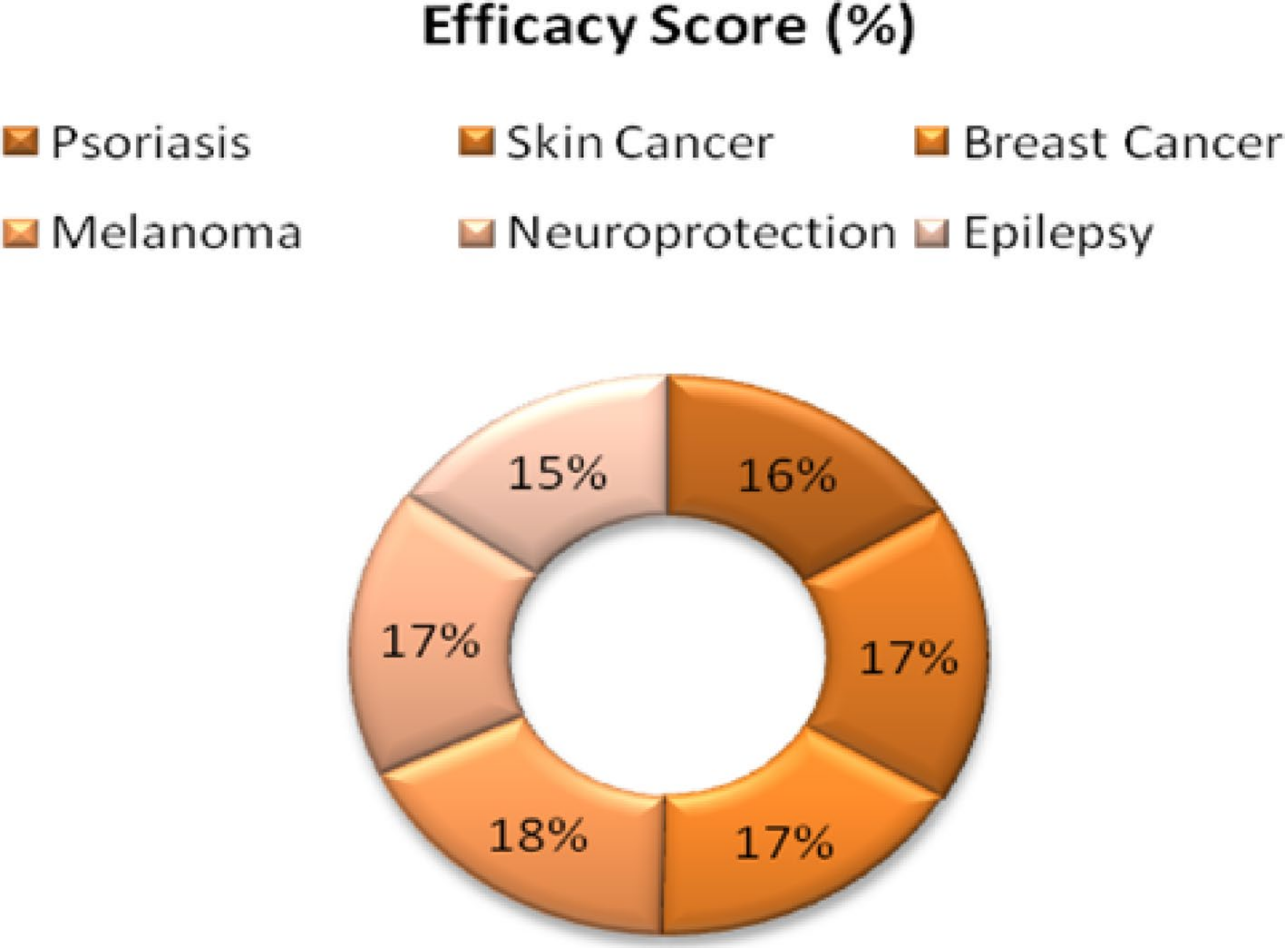
Clinical potential of FTN nanoformulations across diseases

FTN, a flavonoid with bioactive properties, has been of interest due to its anti-inflammatory, anticancer, and neuroprotective activities. However, its limited solubility and bioavailability have required the formulation of nanoformulations that enhance its clinical potency in disease states such as psoriasis, cancer, and neurodegenerative disease states (Table 4). The clinical potential of FTN nanoformulations across diseases is summarized in Fig. 5. In PsO, chronic skin inflammation and FTN nanoformulations have been found to have encouraging results. Chamcheu et al. proved that FTN-polymer nanoparticles suppressed the PI3K/Akt/mTOR and MAPK pathways and suppressed keratinocyte proliferation and inflammation [20]. Likewise, Roy et al. established that dual targeting of mTOR/IL-17A and autophagy with FTN suppressed psoriasis-like inflammation in a mouse model and showed promise of translation in dermatology [21].

**Table 4:** Clinical Applications of FTN Nanoformulations

Disease	Mechanism of action	Nanoformulations used	Findings	References
Psoriasis	Inhibits PI3K/Akt/mTOR and MAPK pathways and reduces keratinocyte hyperproliferation	Polymeric nanoparticles	Reduced inflammation and skin thickening in psoriasis models	[20]
Psoriasis	Targets mTOR/IL-17A and autophagy pathways	Lipid nanoparticles	Reduced inflammation and lesion severity in animal models	[21]
Skin cancer & Psoriasis	Enhances skin penetration, inhibits melanoma cell proliferation	Binary ethosomes	Improved skin retention and drug absorption, reducing tumor growth and inflammation	[93]
Breast cancer	Increases cytotoxicity against tumor cells promotes apoptosis	Polymeric nanoparticles	Controlled release, enhanced drug stability, and improved anticancer effects	[94]
Melanoma & Skin cancer	Enhances drug bioavailability, induces tumor apoptosis	Lipid-based nanocarriers	Significant tumor size reduction in melanoma models	[95]
Neuroprotection (Alzheimer’s)	Reduces oxidative stress and neuroinflammation and improves cognitive function	Lipid nanocarriers	Significant neuroprotective effects in Alzheimer’s disease models	[96]
Epilepsy	Reduces neurobehavioral deficits, modulates ROS/TNF-α pathways	Chitosan nanoparticles	Improved seizure control and reduced brain inflammation	[91]

Furthermore, Chaurasiya et al. (2024) established that FTN-delivered binary ethosomes promoted percutaneous penetration, suppressed inflammation and inflammation-associated markers, and found application in treating melanomas and PsO alike [93]. In cancer treatment, FTN nanoformulations have superior bioavailability, regulated drug release, and higher cytotoxicity against the cells of the tumors. Studies show FTN-encapsulating polymer nanoparticles provided superior anticancer potency in breast, prostate, and oral cancer models [97]. Furthermore, Gupta et al. established the application of FTN-loaded lipidic nanocarriers that showed superior improvement in the suppression of tumors and the bioavailability of the drug in models of melanomas. Further, they established the application of FTN in oncology treatment in the field of oncology [95].

FTN nanoformulations have also been examined in neuroprotection against Parkinson’s and Alzheimer’s. Burlec et al. established that FTN nanoparticles had a pronounced alleviation of neuroinflammation and oxidative stress in models of Alzheimer’s disease and envisioned a future use in neurodegenerative therapy [96]. In addition, FTN-loaded chitosan nanoparticles developed by Mahawar et al. relieved neurobehavioral deficits in models of epilepsy-induced mice and demonstrated the promise of future use in neurological disease [91]. Despite promising preclinical findings, several issues prevail in translating FTN nanoformulations into the clinic. Among them are the barriers to regulatory approval, such as the nonexistence of standard FTN nano-drug formulation and large-scale manufacturing procedures, the prohibitive cost, and the technical sophistication of synthesizing nanoparticles that prevent mass application. In addition, long-term evaluations of the safety of FTN nano-drug formulation must be performed through large-scale clinical trials to determine toxicity, pharmacokinetics, and dosing optimization strategies. Still, the development of nanotechnology, target drug delivery, and combination treatments advances the clinical promise of FTN nano-drug formulation toward future medicinal applications.

## Challenges and opportunities

**Fig. 6:**
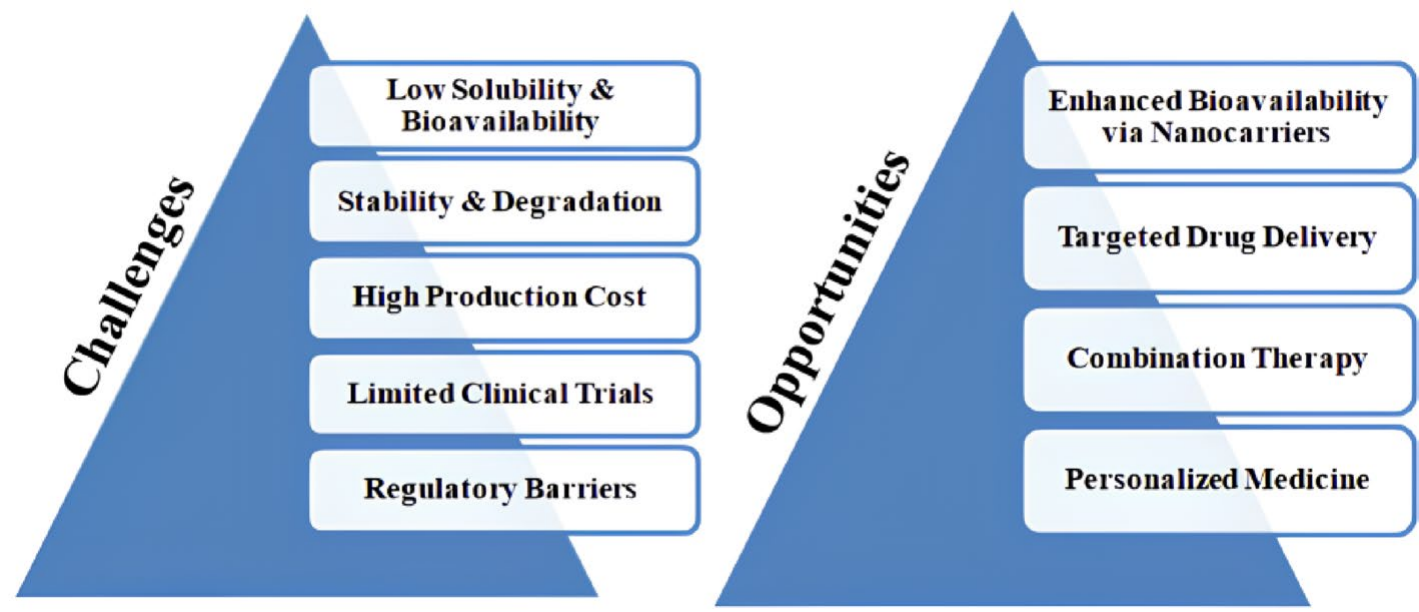
Challenges and opportunities of FTN nanoformulations

FTN nanoformulations face many issues, including low solubility, poor bioavailability, stability, and mass production [98] (Fig. 6). Even with nanoencapsulation techniques, including liposomes, nanoemulsions, and polymeric nanoparticles, achieving maximal solubility and controlled drug release become problematic. The stability of FTN against oxidation [99], susceptibility to pH [100], and enzymatic degradation [98] also become issues of long-term storage and transport. Large-scale production becomes a significant problem. The high expense of synthesizing nanoparticles, batch-to-batch variation, and complex manufacturing processes discourage mass production. The limited clinical testing, the problem of FDA approval, and safety issues also hinder its clinical translation. Despite such issues, nanotechnology-empowered innovations promise a lot. Polymeric nanoparticles and lipidic preparations with a higher bioavailability and controlled drug discharge make FTN a useful drug-targeting option that enhances the scope of the drug. Combining FTN with quercetin, curcumin, and resveratrol enhances the drug’s anticancerous, neuroprotectant, and anti-inflammatory activities. The area of individualized medication forms an ample opportunity space. With the help of innovative drug-delivery systems, liposomes, and PEGylated nanoparticles enhance the drug at the disease site with minimal side effects. Prospects include the formulation of FTN-derived nanomedicines with the aid of future clinical trials, economical mass-scale preparation, and enhancement of the targeting abilities of the drug.

## Conclusion

Herbal medicines have been utilized for centuries to improve the health and welfare of humanity. Because of its pleiotropic pharmacological properties, FTN, a natural polyphenol flavonoid, presents activities against various life-threatening diseases, including cancer and neurological and cardiovascular disorders. FTN has anti-inflammatory, antioxidant, and immunomodulatory properties and shows promise for managing PsO. The major limitation allied with the delivery of FTN is its poor biopharmaceutical properties, such as low aqueous solubility, high lipophilicity, and extensive first-pass metabolism. However, it has been found to possess oral bioavailability of almost 44%.

Preclinical studies suggest that FTN can reduce inflammation, control excessive skin cell turnover, and promote skin regeneration. Nanoformulations, such as liposomes and polymeric nanoparticles, enhance FTN’s skin penetration and stability, ensuring it reaches deeper skin layers where PsO lesions occur. While human clinical trials are still limited, preclinical evidence supports FTN’s potential to reduce symptoms like redness, scaling, and itching.

FTN nanoformulations offer a safer, natural alternative to conventional therapies like corticosteroids, providing targeted, long-lasting relief. These compounds may reduce treatment frequency and side effect rates and be combined with standard treatments to maximize effect. Stability of the formulation, scalability issues, and regulatory issues must be addressed before mass availability. In short, FTN nanoformulations promise a great future in treating PsO, but further study and clinical testing will be needed to ensure they are safe and effective. Assuming they succeed, FTN will be an excellent phytoconstituent option for PsO.

## Data Availability

No datasets were generated or analysed during the current study.

## Abbreviations

PsO: Psoriasis
FTN: Fisetin
PDCs: Plasmacytoid dendritic cells
IFN: Interferon
MDCs: Myeloid dendritic cells
TNF: Tumor necrosis factor
TH1: T Helper 1
TH 17: T Helper 17
TH 22: T Helper 22
VEGF: Vascular endothelial growth factor
NF-Kb: Nuclear factor-kappa B
COX2: Cyclooxygenase-2
MAPK: Mitogen-activated protein kinase
SOD: Superoxide dismutase
CAT: Catalase
VEGF: Vascular endothelial growth factor
SLN: Solid lipid nanoparticle
SNEDDS: Self-nanoemulsifying drug delivery system
TRARS: Thiobarbiturc acid reactive substances
ELISA: Enzyme-linked immunosorbent assay
CD: Cyclodextrins
Nrf2: Nuclear factor erythroid 2-related factor 2
mTOR/AKT: Mammalian target of rapamycin (mTOR)/protein kinase B (Akt)
